# Ultrasmall aqueous starch-capped CuS quantum dots with tunable localized surface plasmon resonance and composition for the selective and sensitive detection of mercury(ii) ions

**DOI:** 10.1039/c9ra09306k

**Published:** 2020-04-07

**Authors:** S. Irudhaya Raj, Adhish Jaiswal, Imran Uddin

**Affiliations:** Department of Chemistry, Indira Gandhi National Tribal University Amarkantak MP India adhish.jaiswal@igntu.ac.in; Aligarh Muslim University Aligarh India

## Abstract

Ultrasmall starch-capped CuS quantum dots (QDs) with controllable size were chemically fabricated in an aqueous medium. The phase of the CuS QDs was confirmed *via* X-ray diffraction (XRD), whereas the characteristic localized surface plasmon resonance (LSPR) peak in the near-infrared (NIR) region was measured using UV-Vis spectroscopy. Transmission electron microscopy and high bandgap analysis confirmed the formation of ultrasmall CuS QDs in the size range of 4–8 nm. CuS QDs have been used for the selective and sensitive detection of Hg^2+^ ions through colorimetric and spectroscopic techniques. The selective sensing of Hg^2+^ ions from various metal ions was detected *via* a remarkable change in color, damping in LSPR intensity, significant change in the Fourier-transform infrared spectra and X-ray photoelectron spectroscopic measurements. The mechanism of interaction between the CuS QDs and Hg^2+^ ions has been deeply explored in terms of the role played by the starch and the reorganization of sulfide and disulfide bonds to facilitate the access of Hg^2+^ ions into the CuS lattice. Finally, an intermediate Cu_2−*x*_Hg_*x*_S nanostructure resulted in the leaching of Cu^+^ ions into the solution, which were further recovered and reused for the formation of fluorescent Cu_2_S nanoparticles. Thus, the entire process of synthesis, sensing and reuse paves the way for sustainable nanotechnology.

## Abstract 2

Colorimetric response of CuS QDs with mercury ions.

## Graphical abstract

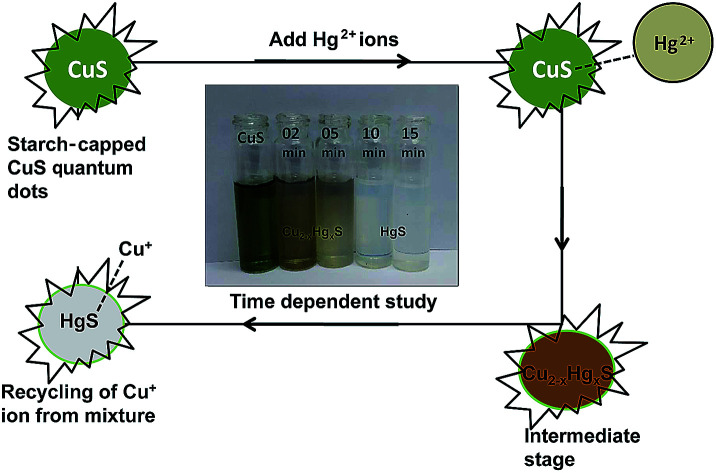

## Introduction

Nature is colorful in the presence of light. Localized surface plasmon resonance (LSPR), the unique phenomenon that manifests in nanoscale materials, originates from the resonant interaction of free charge carriers with incident light. Traditional plasmonic nanoscale metals such as silver,^1^ gold,^2^ and copper^3^ exhibit their corresponding LSPR in the visible region. LSPR observed in metal nanostructures can be slightly tuned in terms of size, shape, free electrons and the surrounding medium. In recent years, semiconductors exhibiting LSPR in the near or mid infra-red (IR) spectral region have drawn considerable attention due to their tunable plasmonic properties.

Semiconductor-plasmonic copper sulfide (CuS) compounds are of interest due their key advantages such as stoichiometry, easy availability, tunable LSPR, low toxicity, recyclability and tunable band gap values from 1.2–2.0 eV.^4–6^ CuS exists in different stable stoichiometries from copper rich chalcocite (Cu_2_S) to sulfur rich covellite (CuS) phases. In between these two stable phases, copper sulfide exists in several stable and meta stable phases due to self-doped cation vacancies, often represented as Cu_2−*x*_S, with very small *x* values.^7^ Cu_2_S, acts as an intrinsic semiconductor, and exhibits no LSPR due to the absence of free carriers. However, stoichiometric CuS, acts as a p-type semiconductor because of Cu vacancies in its lattice and exhibits near infrared (NIR) LSPR in the wavelength range of 900–1200 nm due to the high concentration of free carriers.^6,7^ From previous studies it is evident that the NIR LSPR property of CuS can be tuned by cation exchange,^8,9^ varying the composition, size and crystal structure,^10,11^ oxygen exposure and surface ligands,^12^ temperature,^13^ and phase transition.^14^ Hence, the tunable optical phenomena of CuS open up the biological window in the NIR spectral region for its potential applications in drug delivery,^15^ photothermal therapy^16^ and therapeutics.^17^

Insights into the cation-exchange reactions of CuS are fascinating and intriguing for the research community. Xie *et al.* reported that in a mild reducing environment, Hg^2+^ ions exchange with CuS nanocrystals (NCs) to form HgS, Cu_2−*x*_S/HgS and Cu_2−*x*_S.^8^ Wolf *et al.* compared the difference in damping, shifting and stability of LSPR in Cu_1.1_S nanoparticles, on incorporating Cu^+^ and Ag^+^ ions.^9^ Stam *et al.* reported that Cu^+^ ions intercalation leads to permanent phase transition from CuS to Cu_2_S NCs, whereas Li^+^ ions interaction leads to reversible transition between CuS and CuLiS structures.^18^ Recently, Liu *et al.* reported the reversible nanoscale interconversion from CuS to Cu_2_S nanocrystals (NCs) and *vice versa*.^14^ It is well known that Hg^2+^ ions have strong affinity towards sulfur ligands and sulfur anions (S^2−^) due to soft–soft interactions.^19,20^ Hence, CuS nanostructures (NSs) with an interesting inherent cation exchange nature, unique optical properties and high affinity for S^2−^ ions, can be exploited to develop a selective, sensitive and rapid metal ion sensing platform.

Over the past few years, researchers have fabricated zero-dimensional (0D), one-dimensional (1D), two-dimensional (2D) and three dimensional (3D) CuS NSs, such as nanodots,^21^ nanocrystals,^8^ thin films,^22^ nanotubes,^23^ nanoplates,^24^ nanosheets,^25^*etc.* These CuS NSs have been exploited for potential applications such as bioimaging,^26^ photocatalysis,^27^ nanoelectronics,^28^ and theranostics.^29^ Currently, there are various methodologies to fabricate CuS NSs, such as sonoelectrochemical,^30^ hydrothermal,^30^ solventless thermolysis,^30^ mechanochemical,^31^ microwave,^32^ hot-injection,^14^ and cation-exchange reaction^33^ methods. However, in most of these reports CuS NSs were prepared using expensive solvents, strong surfactants, and at high temperature and pressure.^34^ Moreover, most of these methods were used to prepare CuS NSs with a size greater than 10 nm and unfortunately, that are insoluble or have low solubility in water. Moreover, insoluble CuS has practical limitations in the environmental field, since sensing studies are carried out in water or organic solvents or a mixture of organic solvents/water.^35^

As is known, the size reduction of nanomaterials into 0D quantum dots can cause significant alteration in their physical and chemical properties due to a quantum confinement effect. Shuang *et al.* synthesized stable water soluble amorphous and crystalline CuS QDs with average diameters of 2.7 and 3.3 nm, respectively, and exploited their properties in the anti-proliferation of human cancer cells.^17^ Therefore, it is vital to develop a simple route for the preparation of CuS QDs in water. Over the past few years, QDs have been widely used in the detection of transition metal ions^36^ and heavy metals ions.^37^ Based on their tunable optical properties, cation exchange reactions, sulfur rich nature and quantum size effect we hypothesize that CuS QDs might function as a novel Hg^2+^ ion sensor, with distinct advantages over conventional analytical techniques and other nanomaterial-based sensors in terms of cost, solubility and environmental benignity.

Herein, we present a facile strategy for the synthesis of plasmonic CuS QDs in aqueous medium using a bottom-up approach. Starch, a natural biodegradable polymer, was used as both a reducing and capping agent. The size of the CuS QDs was controlled to below 10 nm by adjusting the reaction parameters. The as-prepared CuS QDs show the selective and sensitive sensing of Hg^2+^ ions in water. Furthermore, we speculate a possible mechanism for the selective sensing of Hg^2+^ ions using the CuS QDs. Moreover, the novel sensing platform provides multiple signal output in terms of color, composition, wavelength and intensity, enhancing its reliability in metal ion detection. Finally, from our reuse experiments, we found that Cu^+^ ions released into the solution can be recovered to synthesize Cu_2_S NSs. Thus, the whole experimental process of synthesis, Hg^2+^ sensing and reusability is an environmentally friendly process that can be used toward sustainable nanotechnology.

## Materials and methods

All of the chemicals were of analytical grade with a purity of more than 98%, purchased from Merck Ltd. The aqueous solutions were prepared with double distilled water (DW) for all of the experiments.

## Synthesis of CuS QDs

Briefly, a transparent starch solution was first prepared by mixing 2 g of starch in 250 ml of DW and the resultant turbid white solution was heated to 70–80 °C under stirring for 15 min. Next, the hot transparent starch solution was used to prepare 1 mmol of CuCl_2_ and Na_2_S solutions separately. Thereafter, the resultant starch mixed CuCl_2_ and Na_2_S solutions were heated to 70 °C under magnetic stirring for 10 min separately. Then, the as prepared hot Na_2_S-starch solution was added dropwise to a hot CuCl_2_-starch solution under vigorous stirring at 50 °C, under a N_2_ atmosphere. After cooling to room temperature, the as-prepared green solution of the CuS QDs was used for further instrumental characterization and experiments.

## Results and discussion

**Fig. 1:**
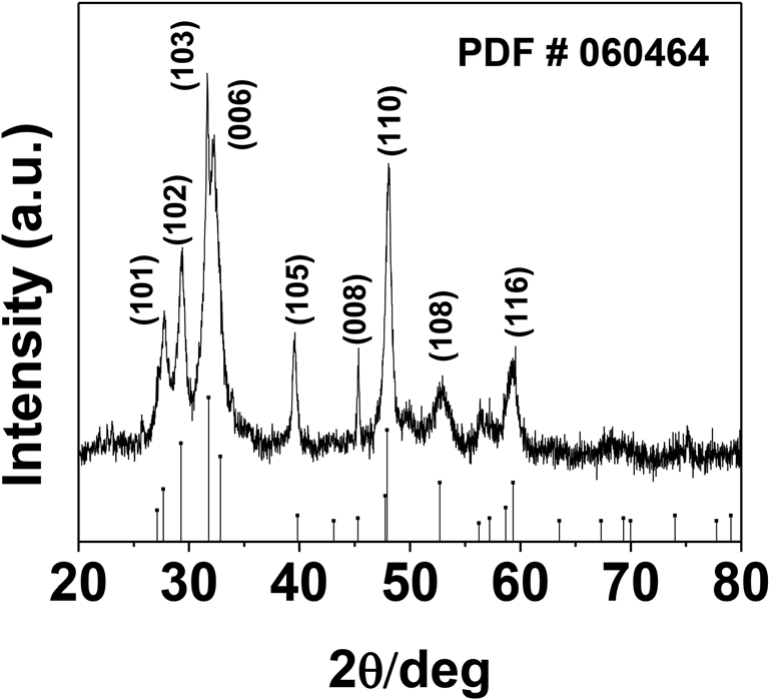
The room temperature XRD pattern of CuS QDs with reference to JCPDS card no. 060464.

The phase and morphology of the as-synthesized starch-capped CuS QDs were characterized by XRD, recorded using a PANalytical X'PERT PRO instrument equipped with an iron-filtered Cu-Kα radiation source (*λ* = 1.5406 Å) in the 2*θ* range of 20–80°, with a step size 0.02. The particle size of the CuS QDs was very small, therefore the sample was dried and calcined (at 110 °C) and then the diffraction pattern was obtained. The phase and purity of the sample were confirmed according to the reference taken from JCPDS card no. 060464. The XRD patterns (Fig. 1) show peaks corresponding to the (103), (006), (110) and (102) planes, indicating that the fabricated sample is hexagonal crystalline CuS QDs.

**Fig. 2:**
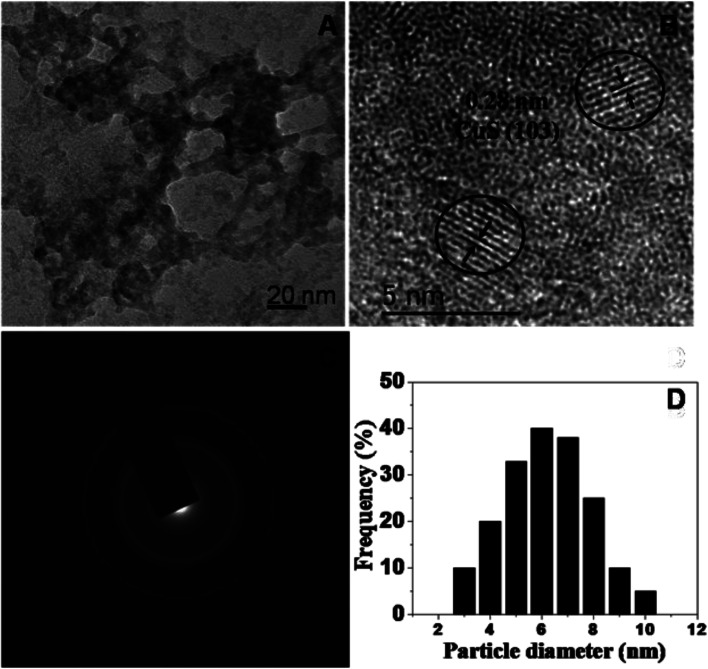
(A and B) TEM images, (C) the SAED pattern and (D) the average particle size distribution of the CuS QDs.

To investigate the nanostructure and topography, we used a FEI (model Tecnai F30) high resolution transmission electron microscope (HRTEM) equipped with a field emission source operated at 300 KV to image the CuS QDs on a carbon-coated copper TEM grid. The TEM images (Fig. 2, panel A) show that the particles are spherical in morphology with an average size of around 4–8 nm. The particles are not well-dispersed but are fused together to form larger particles. The HRTEM image (Fig. 2, panel B) shows an interplanar spacing of 0.28 nm, which corresponds well to the (103) plane of CuS QDs. The selected area electron diffraction (SAED) pattern (Fig. 2, panel C) shows the polycrystalline nature of the CuS QDs. The particle size distribution (Fig. 2, panel D) of the CuS QDs shows that the average particle size is in the range of 4–8 nm.

**Fig. 3:**
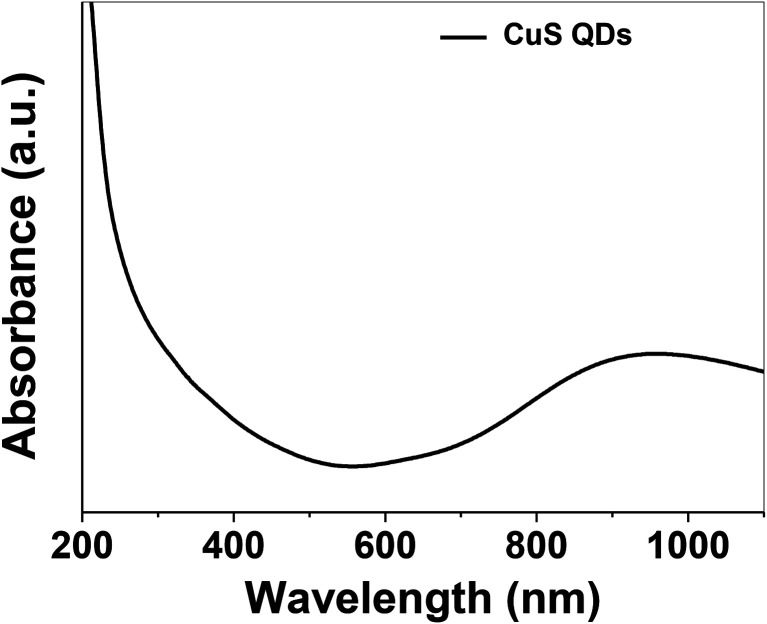
UV-Vis absorption spectrum of the CuS QDs.

The as-synthesized CuS QDs were further characterized by UV-Vis spectroscopy recorded on a UV-Vis spectrophotometer (Shimadzu, UV-1800) in the scan range of 190–1100 nm at a resolution of 2 nm. The UV-Vis absorption spectrum (Fig. 3) of the CuS QDs shows an intense absorption peak in the UV and NIR region with a LSPR peak at approximately 975 nm. The phase purity, reaction parameters and particle size allowed us to tune the LSPR peak of the CuS QDs to 975 nm. However, CuS nanoparticles with a particle size in the range of 10–20 nm exhibited LSPR above 1000 nm.^16^ The blue shift in the absorption spectra and LSPR peak positioning confirm a particle size of less than 10 nm. Moreover, it is known that LSPR is size dependent and red-shifting of peaks occurs with an increase in particle size.^11^

**Fig. 4:**
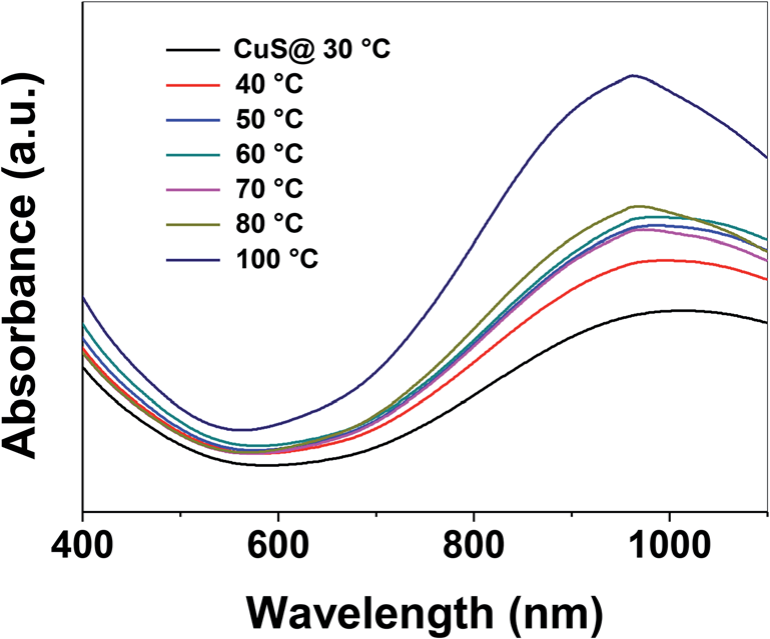
Facile tuning of the LSPR of CuS QDs upon an increase in the temperature.

Control of the optical properties of CuS QDs is fundamental for practical applications. With this in mind, we studied the effect of temperature on the optical behavior of CuS QDs. As the temperature was gradually increased, we observed a series of changes in the LSPR, namely a small blue-shift in the NIR LSPR peak by 15 nm, a distinct change in the shape of the LSPR band, an increase in the LSPR absorption intensity and a narrowing and slight sharpening of the peak line width (Fig. 4). Moreover, these observations confirm that temperature can be used to tune the LSPR of the CuS QDs. In our previous work, we reported that temperature can be used to tune the surface properties of porous silica nanoparticles.^38^ Therefore, the facile tunable LSPR and thermal stability of these CuS QDs opens the door to their use as light harvesting materials and in photothermal therapy.

**Fig. 5:**
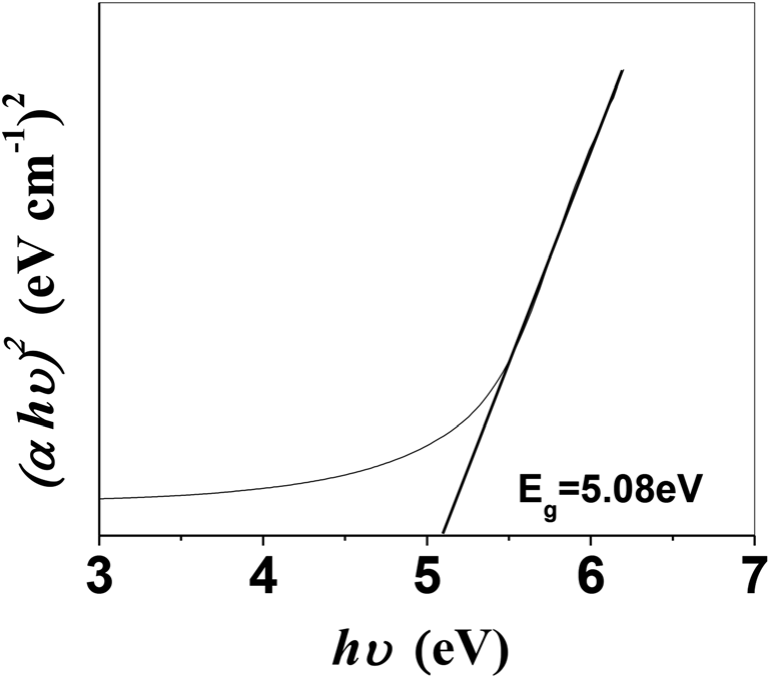
Tauc plot of CuS QDs with a band gap of 5.08 eV.

It is imperative to highlight the energy band structure, which is the difference between the bottom of the conduction band (CB) and the highest occupied state in the valence band (VB).^39^ From the absorption spectra, the direct band gap was measured using the following equation:

1 (*αhν*)^*n*^ = *A*(*hν* − *E*_g_)

where *α* is the absorption coefficient, *hν* is the photon energy, *A* is a constant, *E*_g_ is the band gap, and the value of *n* is 2 for a direct transition. To calculate the direct band gap, we plotted a Tauc plot (Fig. 5) between *hν* and (*αhν*)^2^ and from this the direct band gap of the as-synthesized CuS QDs was measured to be 5.08 eV. It is noteworthy that the band gaps calculated from temperature dependent studies were approximately equal to 5.0 eV. The band gap of the bulk material of CuS is 2.0 eV, while quantum confinement can increase the band gap of bulk semiconductors by 1.0 eV.^10^ In the case of CuS QDs, band gap variation depends on both quantum confinement^30,40^ and the Moss–Burstein effect.^30,41^ A recent study showed that CuS NCs in the size range of 3–9.8 nm exhibited a band gap of 4.33 eV, due to a strong quantum confinement effect.^42^ Therefore, in the present work, the higher direct band gap obtained for the ultrasmall CuS QDs can be attributed to a quantum confinement effect and these results further validate the size measurements seen in the TEM images. The overall results demonstrate the success in fabricating plasmonic CuS QDs in aqueous medium, with an ultrasmall particle size (4–8 nm), high thermal stability, tunable LSPR and high band gap. The green colored solution of the as-prepared CuS QDs indicated that they were well dispersed, homogenous and highly stable and showed no sign of aggregation, even after one month of storage at room temperature in the dark.

### Selective and sensitive detection performance of the CuS QDs

**Fig. 6:**
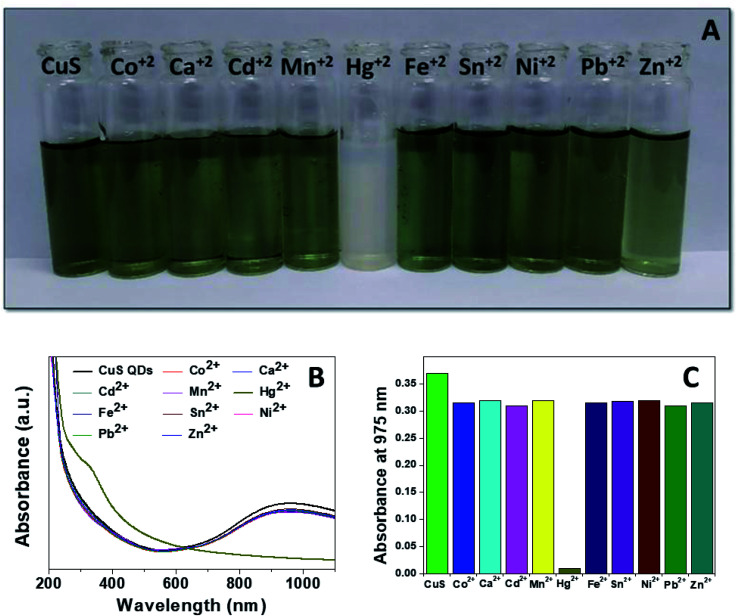
(A) Selective colorimetric detection of CuS QDs with different metal ions. (B) UV-Vis spectra of CuS QDs in the presence of different metal ions. (C) Bar diagram of LSPR responses of the CuS QDs to different metal ions.

**Fig. 7:**
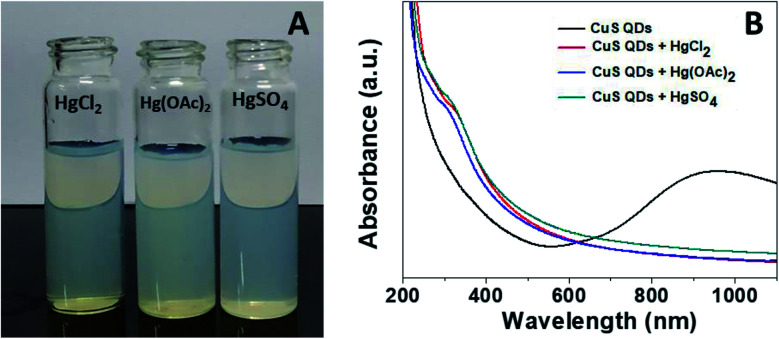
(A) The colorimetric detection performance and (B) UV-Vis spectra of the CuS QDs in the presence of HgCl_2_, Hg(OAc)_2_ and HgSO_4_.

Mercury(ii) ions (Hg^2+^) are a widespread heavy metal pollutant, that have adverse effects on global health and the environment. Therefore, environmental monitoring of aqueous Hg^2+^ ions is in increasing demand to mitigate such global health effects. First, the selective colorimetric detection performance of the CuS QDs for Hg^2+^ ions were assessed in the presence of environmentally interfering cations. Hence, we prepared a solution containing ten common metal ion solutions (Co^2+^, Ca^2+^, Cd^2+^, Mn^2+^, Hg^2+^, Fe^2+^, Sn^2+^, Ni^2+^, Pb^2+^ and Zn^2+^) of 500 μM in concentration. Next, 4 ml of starch-capped CuS QDs were taken up in a series of vials and then 0.5 ml of the abovementioned metal ion solution were added sequentially into each vial. The resulting solution mixture was shaken well and kept at room temperature for 10 min. We observed that the addition of Hg^2+^ ions in CuS QDs resulted in a white colored solution, however, the addition of other metal ions shown no obvious color change (Fig. 6A). To gain insight into the selectivity of the CuS QDs for Hg^2+^ ion detection, we further recorded UV-Vis spectra under the same conditions employed for the colorimetric detection. We observed that the LSPR of the CuS QDs at a wavelength of 975 nm disappeared and a new shoulder at approximately 330 nm appeared on addition of the Hg^2+^ ions, whereas the LSPR was insensitive to the addition of the other metal ions (Fig. 6B and C). Furthermore, the effect of the addition of different mercuric salts such as HgCl_2_, Hg(OAc)_2_ and HgSO_4_ to the CuS QDs also resulted in the appearance of a white colored solution (Fig. 7A) and the disappearance of the LSPR (Fig. 7B). Therefore, this nanosensor shows selective detection performance only for Hg^2+^ ions rather than for other metal cations and anions.

**Fig. 8:**
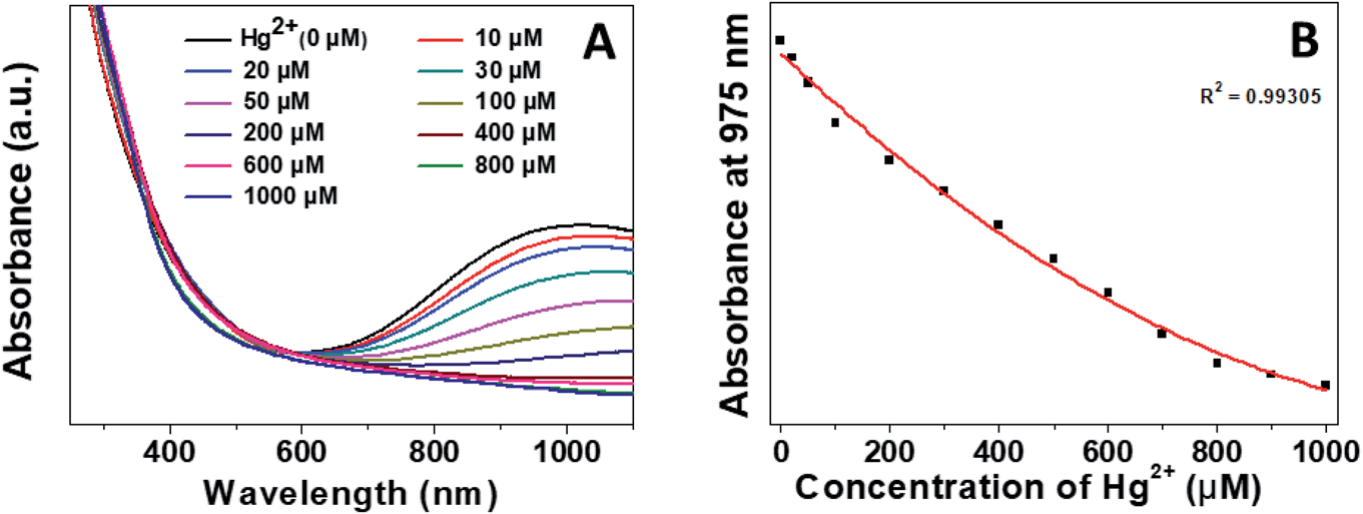
(A) Sensitive detection performance and (B) linear LSPR response of the CuS QDs to different concentrations of Hg^2+^.

The sensitive detection performance of the proposed nanosensor was investigated by UV-Vis absorption spectroscopy. We monitored the LSPR of the CuS QDs at the 975 nm band feature in response to different concentrations of Hg^2+^ ions. The LSPR intensity decreased continually with an increase in Hg^2+^ ion concentration from 0 to 1000 μM (Fig. 8A), with a highly linear correlation coefficient (Fig. 8B).

**Fig. 9:**
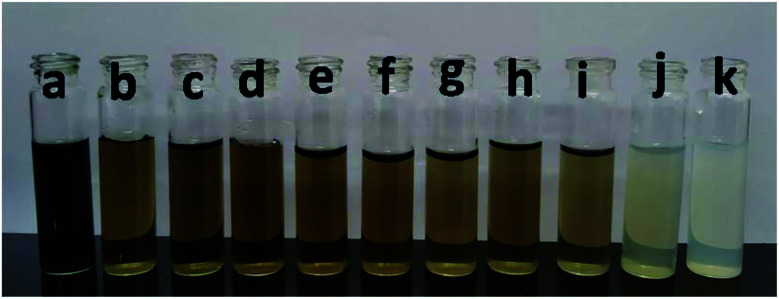
Colorimetric responses upon the addition of different Hg^2+^ ion concentrations to the CuS QDs: (a) 0, (b) 10, (c) 20, (d) 30, (e) 50, (f) 100, (g) 200, (h) 400, (i) 600, (j) 800, and (k) 1000 μM.

Colorimetric sensitivity is one of the key factors for a sensing system. Interestingly, we observed that the naked eye colorimetric responses of the CuS QDs were different against different concentrations of Hg^2+^ ions (Fig. 9). It is clear that the addition of 10 μM of Hg^2+^ ions changes the green colored CuS QDs to brown and upon a further increase in concentration, this brown color diminishes to pale brown and finally to white. Therefore, the limit of detection (LOD) for the Hg^2+^ ions was 10 μM by eye.

**Fig. 10:**
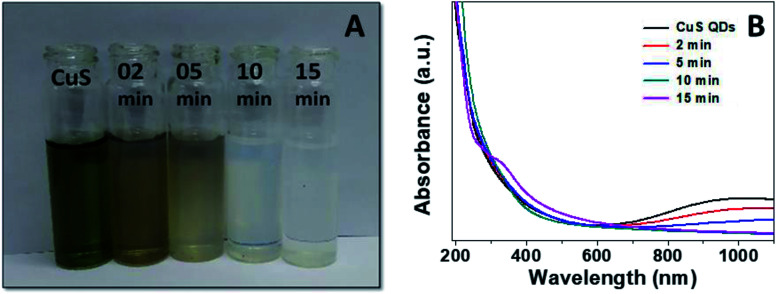
(A) Colorimetric responses and (B) UV-Vis absorption spectra of the CuS QDs to Hg^2+^ ions over time.

Finally, to gain insight into the selectivity and sensitivity of the CuS QDs for Hg^2+^ ion detection, we examined how the color and absorption spectra change with time upon the addition of Hg^2+^ ions (500 μM). We observed a sequence of colorimetric change from green to white *via* brown, pale brown and pale white with an increase in time from 0–15 min (Fig. 10A). Simultaneously, UV-Vis absorption spectra were also recorded under the same conditions to those of the colorimetric responses. From the time-dependent UV-Vis absorption spectra, we observed a sequence of changes, namely a slight red shift with a gradual decrease in LSPR intensity, the disappearance of LSPR, and finally, the appearance of a new shoulder at approximately 340 nm (Fig. 10B). These results demonstrate that the nanosensor platform provides a multiple signal output in terms of color, intensity and wavelength.

It is well known that the target analyte is extremely toxic, therefore the detection limit is significant. At a signal-to-noise ratio of 3, the limit of detection (LOD) for the Hg^2+^ ions was estimated to be 1.95 μM. The limit of detection (LOD) of the proposed colorimetric sensing platform is compared with the other colorimetric sensors reported in the literature in Table 1.

**Table 1:** Comparison of the proposed colorimetric nanosensor platform with other reported colorimetric sensor platforms for the detection of Hg^2+^ ions

Quantitative method	Sensor platform	Linear range	Spectroscopic LOD	Reference
Colorimetry	CuS QDs	10–1000 μM	1.95 μM	Present work
	Au nanoparticles	0.5–5.0 nM	0.2 nM	43
	Cu_2−*x*_Se nanoparticles	0 nM to 10 μM	2.7 nM	44
	Modified polyacrylonitrile fiber	1–10 000 μM	1.0 μM	45
	MoS_2_ nanosheets	25 nM to 2.5 μM	3.5 μM	46
	Copper nanoparticles	0–75 μM	0.11 μM	3
	Silver nanoprisms	0–5.0 μM	3.0 nM	47
	Metal oxo-clusters	0.2–1.4 μM	0.05 μM	48

**Fig. 11:**
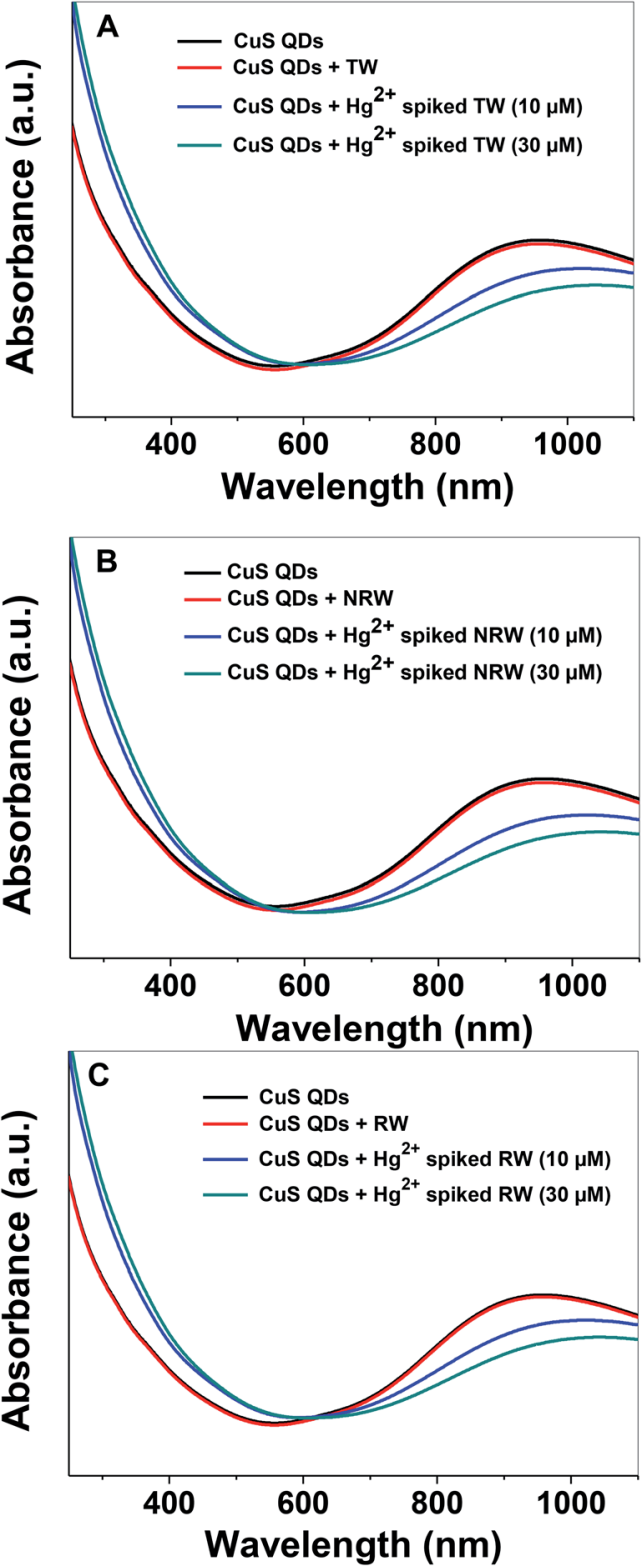
Spectroscopic sensing of Hg^2+^ in (A) TW (B) NRW and (C) RW samples by the CuS QDs.

In order to evaluate the practical applicability of the proposed colorimetric assay for the detection of Hg^2+^ ions, various water samples, (TW: tap water from the IGNTU campus, RW: rain water harvesting pond, IGNTU campus, NRW: Narmada river, India) from regional sources were collected, filtered and tested using a standard addition method. Unspiked water samples and samples spiked with 10 μM and 30 μM of Hg^2+^ were prepared using various water resources. The UV-Vis absorption spectra of the CuS QDs remain almost invariable in the presence of various unspiked water samples (Fig. 11A–C). On the other hand, the Hg^2+^-spiked samples, spiked with either 10 or 30 μM of Hg^2+^, show red shifting with a decrease in the LSPR intensity. These characteristic changes in the absorption signatures validate that the Hg^2+^ ions are definitely detected in various water samples. Furthermore, the 10 μM and 30 μM Hg^2+^ spiked real water sample recovery rates are in the satisfactory range of 65–87% (Table 2). These results demonstrate that the colorimetric sensing platform can potentially be used for monitoring Hg^2+^ ion content in drinking water.

**Table 2:** The proposed Hg^2+^ ion sensing platform being used in various real water samples

Sample	Added Hg^2+^ (μM)	Proposed sensor (μM)	Recovery (%)
TW	0	Not detected	—
	10	8.7	87.0
	30	24.3	81.0
RW	0	Not detected	—
	10	7.8	78.0
	30	22.2	74.0
NRW	0	Not detected	—
	10	7.0	70.0
	30	19.5	65.0

### Evolution: CuS to HgS *via* Cu_2−*x*_Hg_*x*_S NSs

First, we compared two cases to explore the role of capping agent and particle size effect on the sensing of Hg^2+^ ions by the CuS QDs. In the first case, as stated in our present work, the as-synthesized starch-capped CuS QDs exhibited sensing for Hg^2+^ ions. Whereas, in the second case, a CuS precipitate was prepared using the same precursors and reaction conditions as described above in the synthesis of the CuS QDs without a starch solution. Interestingly, addition of Hg^2+^ ions to the CuS precipitate showed no positive colorimetric and UV-Vis spectroscopic changes. These results further support and validate the effect of particle size and capping agent in the CuS QDs for the sensing of Hg^2+^ ions. Next, we studied the interaction of Hg^2+^ ions with the CuS QDs and speculated a possible interaction mechanism and sequential change in composition.

**Fig. 12:**
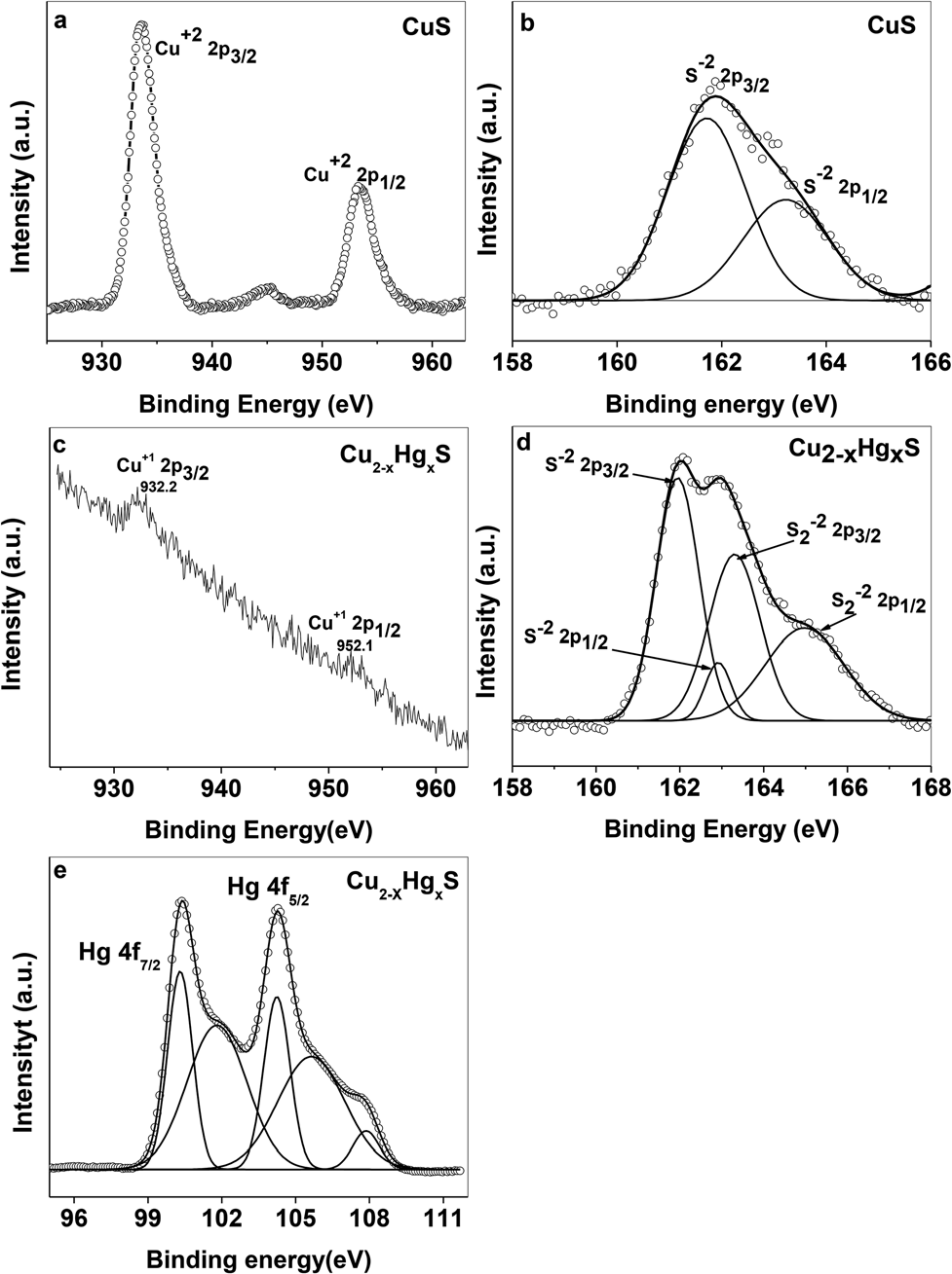
XPS spectra of (a) Cu and (b) S in the CuS QDs, and (c) Cu, (d) S and (e) Hg in the Cu_2−*x*_Hg_*x*_S NSs.

With the aim of investigating the evolution of Cu_2−*x*_Hg_*x*_S from CuS, we carried out X-ray photoelectron spectroscopy (XPS) analysis. For this purpose, the high resolution XPS data was collected by using a VG Microtech, model no ESCA 3000 equipped with an ion gun (EX-05) for cleaning the surfaces. The binding energy resolution was 0.1 eV. The XPS spectra reveal the oxidation state of Cu and S before evolution (Fig. 12a and b) and Cu, Hg and S after evolution (Fig. 12c–e). In the CuS QDs, the doublet peaks in the Cu 2p spectra centered at 933.4 and 954 eV were assigned as Cu^2+^ 2p_3/2_ and Cu^2+^ 2p_1/2,_ respectively (Fig. 12a).^49–51^ The presence of weak peaks at 944.2 eV can be attributed to satellite peaks of Cu^2+^. The feature in the S 2p spectra of CuS and Cu_2−*x*_Hg_*x*_S exhibited several interesting changes during the evolution of Cu_2−*x*_Hg_*x*_S from CuS. The S 2p spectra of the covellite CuS QDs feature doublet peaks at 161.8 and 163.2 eV (Fig. 12b) assigned to S 2p_3/2_ and S 2p_1/2_ respectively, which correspond to a sulfide ion peak as a result of a Cu–S–Cu bonding configuration.^50,52,53^ In comparison, for Cu_2−*x*_Hg_*x*_S two doublet peaks were observed (Fig. 12d). The first doublet peak for sulfide ions has an S 2p_3/2_ component at 161.9 eV and the other S 2p_1/2_ component at 163 eV can be attributed to Cu–S bonding.^50^ The second doublet peak for disulfide ions shows an S 2p_3/2_ component at 163.2 eV and another S 2p_1/2_ component at 164.9 eV that appear as deconvoluted peaks (Fig. 12d), which can be attributed to a Cu–S–S–Hg bonding configuration.^50,52^ The disulfide ion bond energy appears at a higher binding energy than that of the sulfide ion. The presence of another doublet for disulfide bond energy corresponds to the highly nonstoichiometric copper deficient sulfide form. During this process there is a structural transformation and reorganization of the S atoms in the lattice, which facilitates the access of the Hg^2+^ ions into the CuS lattice. Meanwhile, Cu^+^ ions are released from the lattice and Hg^2+^ ions occupy their position, resulting in the formation of Cu_2−*x*_Hg_*x*_S NSs. It is noted that Cu^+^ ions released from the CuS lattice go into solution and can be recovered by filtration. The above proposal was confirmed from the Cu 2p XPS spectra of nonstoichiometric Cu_2−*x*_Hg_*x*_S (Fig. 12c). The XPS spectra confirmed the absence of the Cu^2+^ state and the presence of a small doublet peak at Cu 2p_3/2_ (932 eV) and Cu 2p_1/2_ (952 eV), which can be attributed to the Cu^+^ state. The Hg 4f spectrum also exhibits a doublet feature due to spin orbit splitting and the resultant doublet peaks at 100.33 and 104.23 eV can be assigned to 4f_7/2_ and 4f_5/2_, respectively (Fig. 12e), which correspond to the Hg^0^ state and another doublet peak at 101.81 and 105.67 eV can be attributed to the Hg^2+^ state.^54,55^ Moreover, the Hg^0^ state present on the surface can be attributed to the reducing effect of starch.

During the process of evolution, the CuS QDs exhibited ratiometric, colorimetric, wavelength and intensity responses to varying concentrations of Hg^2+^ ions. The addition of a low concentration of Hg^2+^ ions into the CuS QD solution brought about a colorimetric change (green to pale brown) and composition change (CuS to Cu_2−*x*_Hg_*x*_S and Hg_*x*_S NSs), which was evidenced by the strong red shifting and damping of the LSPR intensity. As the reaction proceeded, further exchange resulted in colorimetric change (pale brown to pale white) and composition change (Cu_2−*x*_Hg_*x*_S to Cu_2_S and HgS), and the formation of Cu_2_S was evidenced by complete disappearance of the LSPR and fluorescence being exhibited in the presence of UV light. Interaction of the Hg^2+^ ions favored the switching of the plasmonic CuS QDs to fluorescent Cu_2_S NSs. Finally, a high concentration of Hg^2+^ resulted in complete cation exchange and the resulting formation of HgS NSs, shown as a colorimetric change (pale white to milky white) and composition change (Cu_2_S to HgS & Hg^0^). The formation of HgS was evidenced by the appearance of a new shoulder at 340 nm and the presence of Hg^0^ was confirmed by XPS studies.^56^ By controlling the addition of Hg^2+^ ions, this method gives additional advantages to shift the LSPR to a desired wavelength and prepare Cu_2−*x*_Hg_*x*_S NSs. This is in agreement with previous studies on the transformation of CuS NCs into Cu_2_S *via* the incorporation of Cu^+^ ions and CuS into Cu_2−*x*_S *via* the incorporation of Hg^2+^ and Cd^2+^ ions.^7,8^ However, the reaction time for transformation in these previous works was 24 h.^7,8^ In our present study, the starch-capped CuS QDs with unique properties reacted with Hg^2+^ ions swiftly and the transformation of CuS to HgS occurred within 15 min.

**Fig. 13:**
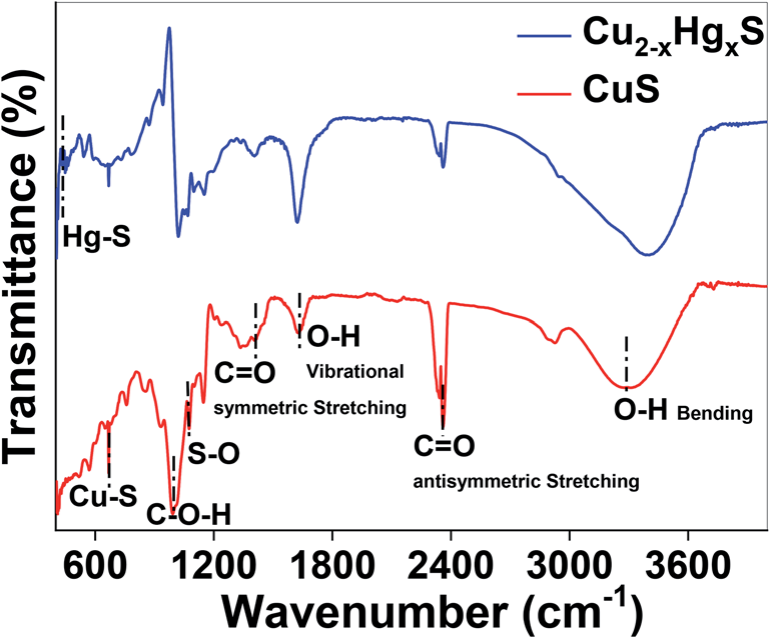
FTIR spectra of CuS and Cu_2−*x*_Hg_*x*_S.

Finally, the transformation of CuS to HgS was corroborated using Fourier-transform infrared (FT-IR) spectroscopy (Thermo Scientific iD7 ATR, Nicolet iS5) over a scanning range of 400 to 4000 cm^−1^ at a resolution of 4.0 cm^−1^ (Fig. 13). It can be observed that by comparing the FT-IR spectra of CuS and Cu_2−*x*_Hg_*x*_S, the characteristic vibrations at 3297 and 1623 cm^−1^ can be assigned to the vibrational and bending modes of OH groups, the peaks at 2360 and 1633 cm^−1^ can be assigned to the hydroxyl bending mode of water molecules, and the peak at 947 cm^−1^ can be assigned to the C–O–H bending vibration of starch, which exhibited a significant change in transmittance after the interaction with the Hg^2+^ ions. The absorption bands between 1200 to 1000 cm^−1^ attributed to the C–O, C–C and C–O–H bending vibrations of starch, significantly changed due the reduction process being carried out by the starch.^57^ The vibrational peaks at 668 and 1074 cm^−1^, assigned to the Cu–S stretching modes and asymmetric valence S=O vibration,^58,59^ decreased after the conversion of CuS to Cu_2−*x*_Hg_*x*_S, confirming the apparent rupturing of Cu–S and S=O bonds. Furthermore, the appearance of a new vibrational peak at 448 cm^−1^, assigned to Hg–S stretching,^60^ of considerable intensity, is an indication that some of the CuS was transformed into HgS. Thus, taken together, the UV-Vis, XPS and FTIR studies support our hypothesis that the CuS QDs were converted into HgS *via* Cu_2−*x*_Hg_*x*_S nanostructures as a result of cation exchange reactions and the reorganisation of the lattices.

## Reuse experiment

Notably, the Cu^+^ ions released into the solution from the exchanged lattices of CuS QDs were used in the formation of Cu_2_S NSs. In a typical recycling experiment, we collected and centrifuged the milky white solution. The supernatant solution containing Cu^+^ ions was slightly warmed to 40 °C and mixed with 1 mmol of hot starch mixed with a Na_2_S solution under a nitrogen atmosphere. The resultant mixture was left undisturbed and a brown colored solution was obtained. The UV-Vis absorption spectra of the resultant solution exhibited no peak or band in the NIR region. Furthermore, the brown solution showed photoluminescence, under a short UV light of 254 nm. As we know, Cu_2_S NSs exhibit no LSPR in the NIR region and the displaying of photoluminescence evidences the success of the recycling process.^18^ Moreover, the results of this study expand our understanding on cation-exchange chemistry and provide a comprehensive approach to synthesize other familiar copper chalcogenides (CuSe, CuTe) and stoichiometric metal sulfides (NiS, FeS and CoS) NSs and QDs.

## Conclusions

We have presented a cost-effective and environmentally friendly approach to synthesize CuS QDs of controllable size in an aqueous medium. Cation exchange reactions, the reorganisation of sulfide and disulfide bonds, and the reducing properties of starch provide a unique set of tools to design a multi-output nanosensing platform for the detection of Hg^2+^ ions in water. The metal ion exchange and sensing mechanism can be used to convert CuS to non-stoichiometric Cu_2−*x*_S NSs with the desired composition. Notably, the recycling experiments enabled us to synthesize Cu_2_S NSs, which further validated the environmentally friendly and sustainable nanotechnology approach. Overall, the CuS QDs have unique tunable optical properties and represent a rapid and feasible method for Hg^2+^ assays.

## Abbreviations

CuS: Covellite
Cu_2_S: Chalcocite
QDs: Quantum dots
NCs: Nanocrystals
NSs: Nanostructures
LSPR: Localized surface plasmon resonance
UV: Ultraviolet
UV-Vis: Ultraviolet-visible
NIR: Near-infrared
CB: Conduction band
VB: Valance band
LOD: Limit of detection

## Conflicts of interest

There are no conflicts of interest.

## Supplementary Material
